# β-Hydroxy-β-Methylbutyrate (HMB) Supplementation Prevents Bone Loss during Pregnancy—Novel Evidence from a Spiny Mouse (*Acomys cahirinus*) Model

**DOI:** 10.3390/ijms22063047

**Published:** 2021-03-17

**Authors:** Ewa Tomaszewska, Janine Donaldson, Jakub Kosiński, Piotr Dobrowolski, Agnieszka Tomczyk-Warunek, Monika Hułas-Stasiak, Krzysztof Lamorski, Dorota Laskowska-Woźniak, Siemowit Muszyński, Rudolf Blicharski, Tomasz Blicharski

**Affiliations:** 1 Department of Animal Physiology, Faculty of Veterinary Medicine, University of Life Sciences in Lublin, Akademicka St. 12, 20-950 Lublin, Poland; 2 School of Physiology, Faculty of Health Sciences, University of the Witwatersrand, 7 York Road, Parktown, Johannesburg 2193, South Africa; janine.donaldson@wits.ac.za; 3 Department of Rehabilitation and Orthopaedics, Medical University in Lublin, Jaczewskiego St. 8, 20-090 Lublin, Poland; kuba.kosinski@gmail.com (J.K.); a.tomczykwarunek@gmail.com (A.T.-W.); blicharskirudolf@gmail.com (R.B.); blicharski@vp.pl (T.B.); 4 Department of Functional Anatomy and Cytobiology, Faculty of Biology and Biotechnology, Maria Curie-Sklodowska University, Akademicka St. 19, 20-033 Lublin, Poland; piotr.dobrowolski@umcs.lublin.pl (P.D.); monhul@o2.pl (M.H.-S.); 5 Bohdan Dobrzański Institute of Agrophysics of the Polish Academy of Sciences, Doświadczalna St. 4, 20-290 Lublin, Poland; k.lamorski@ipan.lublin.pl; 6 Department of Biophysics, Faculty of Environmental Biology, University of Life Sciences in Lublin, Akademicka St. 13, 20-950 Lublin, Poland; dorota.laskowska.wozniak@gmail.com (D.L.-W.); siemowit.muszynski@up.lublin.pl (S.M.)

**Keywords:** β-hydroxy-β-methylbutyrate, pregnancy, bone, spiny mouse model

## Abstract

The aim of this study was to determine the effects of ß-hydroxy-ß-methylbutyrate (HMB) supplementation during pregnancy on postpartum bone tissue quality by assessing changes in trabecular and compact bone as well as in hyaline and epiphyseal cartilage. The experiment was carried out on adult 6-month-old female spiny mice (*Acomys cahirinus*) divided into three groups: pregnant control (PregCont), pregnant HMB-treated (supplemented with 0.02 g/kg b.w of HMB during the second trimester of pregnancy, PregHMB), and non-pregnant females (NonPreg). Cross-sectional area and cortical index of the femoral mid-shaft, stiffness, and Young modulus were significantly greater in the PregHMB group. Whole-bone mineral density was similar in all groups, and HMB supplementation increased trabecular number. Growth plate cartilage was the thinnest, while the articular cartilage was the thickest in the PregHMB group. HMB supplementation increased the content of proteoglycans in the articular cartilage and the percentage of immature collagen content in metaphyseal trabeculae and compact bone. In summary, dietary HMB supplementation during the second trimester of pregnancy intensifies bone metabolic processes and prevents bone loss during pregnancy.

## 1. Introduction

Human skeletal diseases are a common problem that contributes to social and economic burdens. Currently, the most common metabolic disease of bone tissue is reduction in bone mass—leading to the development of osteopenia and osteoporosis. As a systemic disease, it is characterized by impaired microarchitecture of skeletal bone tissue and reduced bone strength, which increases the risk of fractures, including low-energy fractures. During pregnancy, mammalian fetuses undergo particularly intense growth and development processes, leading to an increased demand on the mother for nutrients and building materials. During this period, the administration of biologically and physiologically active substances to mothers may have a significant influence on skeletal metabolic processes. The prevalence of bone diseases and bone-disease-related issues in humans highlights the need for new and effective methods of prevention and possible treatment of disorders related to the skeletal system, as well as for studies on the physical and nutritional factors that may affect skeletal tissue remodeling and homeostasis.

For many years, research has been conducted on the use and beneficial effects of ß-hydroxy-ß-methylbutyrate (HMB). HMB is an endogenous metabolite of leucine, an essential amino acid that has a branched aliphatic side chain. Leucine transamination leads to the formation of alpha-ketoisocaproic acid (KIC), which then, together with the participation of the cytoplasmic enzyme KIC-dioxygenase, is converted into HMB. HMB is then converted into beta-hydroxy-beta-methylglutaryl-Co-A (HMG-CoA) mainly in the cytoplasm of muscle and liver cells, where it can be used in the synthesis of cholesterol [1]. HMB has been shown to protect muscles against damage and even accelerate their regeneration. HMB supplementation has shown positive results in terms of the growth and functionality of muscle mass, due to its anabolic and anti-catabolic properties and an extended half-life in the blood, compared to leucine. In addition, HMB appears to be able to increase the benefits of planned rehabilitation in the elderly or after prolonged immobilization [1,2]. The influence of HMB on the wound-healing process and on bone fractures in both humans and laboratory animals has also been confirmed. There is promising evidence that HMB may improve bone density and cognition, and reduce abdominal obesity [3,4,5,6]. HMB supplementation has also been associated with lower blood pressure and low-density lipoprotein cholesterol levels in people with hypercholesterolaemia [7,8,9,10]. The multiple beneficial effects of HMB make it widely available as a dietary supplement for athletes. To date, no adverse effects have been reported in conjunction with HMB supplementation, including annual supplementation.

Although research on HMB has been going on for almost 30 years, to date there aren’t enough reliable studies to indicate whether HMB is safe to use during pregnancy. This gap in the knowledge of the effects of HMB during pregnancy makes this dietary supplement, which is generally considered safe, not recommended for use during pregnancy [9,11]. Available data in animal models (mostly pigs) suggests that HMB supplementation during pregnancy can result, when supplemented at certain doses during selected periods of pregnancy, in the prenatal programming of several organs and tissues within the offspring, including muscle tissue [12], bone tissue [13], and brain tissue [14]. Prenatal supplementation of HMB during pregnancy has been shown to have a positive effect on offspring birth weight, as well as on the morphological, geometrical, and mechanical bone parameters and daily postnatal gains of offspring [15,16,17]. Prenatal administration of HMB to sows during pregnancy had a positive effect on the development of the skeletal system in offspring, by increasing the mechanical and osteometric properties of the bones [13].

Spiny mice (*Acomys cahirinus*) have been used in numerous previous studies, including as a diet-induced type 2 diabetes model, a dielectric rhythm model, a late-pregnancy development model, and a model of parental behavior. Recently, spiny mice have emerged as a new animal model for the study of evolution, development, and regeneration. The long gestation period of the mice, which lasts between 38 and 39 days, ensures that organogenesis is largely complete by the end of gestation and spiny mice pups are more developed at birth compared to other laboratory rodents (mice and rats). Together with the fact that the average litter size of the spiny mouse is 2–4 pups, the spiny mice are an ideal animal model for studies on human pregnancy, perinatal biology, and development [18,19,20,21,22]. They also show greater regenerative abilities than other species of laboratory mice, including for pregnancy-associated disorders [23,24,25,26,27].

Based on previous results, we hypothesized that HMB supplementation during pregnancy may have a positive effect, not only on the offspring, but also on the pregnant dam. Taking into account the influence of pregnancy on bone health, it seems reasonable to determine the effects of HMB supplementation during pregnancy on the mineralization and mechanical properties of the skeletal system of the pregnant dam.

Therefore, the objective of this study was to determine the effects of HMB supplementation on the bone structure and metabolism in a pregnant animal model. In doing so, the present study examined (i) the general bone health (mineralization, mechanical endurance), and (ii) the effects of HMB supplementation, during the second trimester of pregnancy, on trabecular bone histomorphometry (to examine whether HMB protects against pregnancy-induced bone loss) of the spiny mice. In addition, changes in articular cartilage morphology were studied after HMB supplementation. Together, these measurements should provide us with fundamental information on the safety and outcomes of HMB supplementation during pregnancy, in relation to the general bone metabolism of the pregnant dams.

## 2. Results

### 2.1. Body Weight

The length of the gestational period (39–40 days), as well as the number of newborn pups (between two and five), was not different between the HMB-treated mice and the pregnant control group. There were no stillborn pups.

The body weights (49.5 g ± 1.21 g) of the pregnant control females and of the pregnant HMB females (46.5 g ± 2.0 g) (as recorded immediately after delivery of the pups) did not differ themselves. The body weight of the females at the age of 6 months (43.8 g ± 4.0 g) was not significantly different from that of the pregnant control females that gave birth.

### 2.2. Bone Analysis

**Figure 1:**
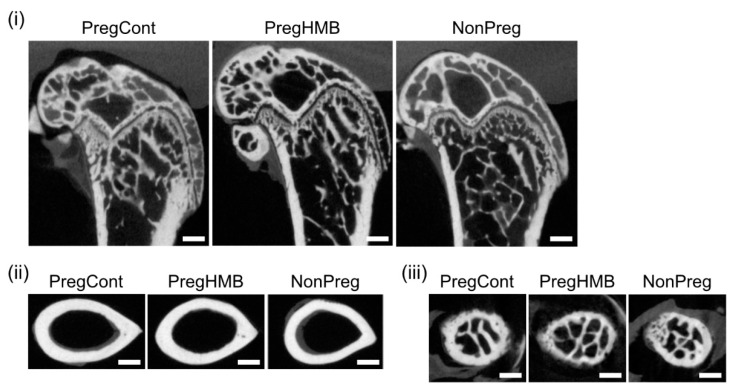
Representative micro-CT images of sagittal sections of the right femur distal diaphysis (**i**), transverse sections of the femur mid-shaft (**ii**), and transverse sections of the femoral neck (**iii**) from pregnant (control and supplemented with β-Hydroxy β-methylbutyrate (HMB)) and non-pregnant female spiny mice. PregCont—control pregnant females (not receiving HMB); PregHMB—pregnant HMB females (receiving HMB at a dose of 0.02 g/kg b.w. during the middle trimester of pregnancy); NonPreg—non-pregnant females. Scale bars represent 500 µm.

Table 1 shows the osteometric, mechanical, and densitometric parameters of the femora from control pregnant females (PregCont), pregnant HMB-supplemented females (PregHMB), and non-pregnant females (NonPreg). The weight and length of femora from females that gave birth were not significantly different between groups, but bones of females from the NonPreg group were significantly lighter and shorter compared to the PregCont group. Femoral stiffness was significantly higher in the pregnant females following HMB supplementation compared to the PregCont group. The femoral stiffness of the non-breeding females was significantly lower compared to the PregCont group. The bending moment and the cross-sectional moment of inertia were significantly lower in the non-breeding females compared to the PregCont group. The cross-sectional area of the femoral mid-shaft in the group of pregnant females supplemented with HMB was significantly larger in comparison to that observed in the PregCont group, and lower in the NonPreg group compared to the PregCont group (Figure 1, ii). The mean relative wall thickness (MRWT) and cortical index of the femora were significantly higher in the group of pregnant females that were supplemented with HMB during pregnancy compared to the PregCont group. Other changes were not observed.

Table 2 shows the material parameters of the femora from control pregnant females (PregCont), pregnant HMB-supplemented females (PregHMB), and non-pregnant females (NonPreg). The Young’s modulus was significantly greater in the group of pregnant females supplemented with HMB during pregnancy. Elastic stress and ultimate strain of the femora of non-breeding females was higher compared to that of the groups of the control pregnant females. Ultimate stress of the femora was significantly lower in the PregHMB group to compared to the PregCont group.

### 2.3. Histomorphometry of Trabecular Bone

Table 3 shows the histomorphometric parameters of the femoral trabeculae from control pregnant females (PregCont), pregnant HMB-supplemented females (PregHMB), and non-pregnant females (NonPreg). The percentage of trabecular bone (bone volume density, BV/TV) in the distal epiphysis in the group of pregnant females that gave birth and were supplemented with HMB during pregnancy was significantly higher compared to that observed in the PregCont group. Non-breeding females showed significantly lower BV/TV. Trabecular thickness of the distal epiphysis was significantly lower in the group of pregnant HMB-supplemented females compared to the PregCont group. Trabecular thickness in the NonPreg group also was lower compared to the PregCont group. The trabecular space of the distal epiphysis was significantly greater in the group of non-breeding females and significantly lower in the group of pregnant females that were supplemented with HMB during pregnancy, compared to the PregCont group. Pregnant females that were supplemented with HMB during pregnancy had significantly more trabeculae at the distal epiphysis compared to the PregCont group. Specific bone surface at the distal epiphysis was significantly lower in the group of pregnant females supplemented with HMB during pregnancy compared to the PregCont group.

BV/TV in the distal metaphysis in the PregHMB group was significantly higher compared to that observed in the PregCont group. Trabecular thickness of the distal metaphysis was significantly higher in the NonPreg group compared to the PregCont group. The trabecular space was significantly greater in the group of non-breeding females and significantly lower in the PregHMB group compared to the PregCont group. Pregnant females that were supplemented with HMB during pregnancy had significantly more trabeculae, while the NonPreg group had less trabeculae at the distal metaphysis compared to the PregCont group. Specific bone surface was significantly lower in the PregHMB group, while it was higher in the NonPreg group compared to the PregCont group

BV/TV in femoral neck in the NonPreg group was significantly higher compared to that observed in the PregCont group. Trabecular thickness was significantly lower in the PregHMB group compared to the PregCont group. Changes in the trabecular space were not observed in femoral neck. Pregnant females that were supplemented with HMB during pregnancy and females that did not give birth had significantly more trabeculae compared to the PregCont group. Specific bone surface was significantly lower in the PregHMB group compared to the PregCont group.

### 2.4. Analysis of Epiphyseal and Articular Cartilage

The histomorphometric analysis of the growth plate and articular cartilage of femora from control pregnant females (PregCont), pregnant HMB-supplemented females (PregHMB), and non-pregnant females (NonPreg) is presented in Table 4.

The total thickness of the growth plate cartilage in the PregHMB group was significantly lower compared to the PregCont group. The thickness of zone II was significantly greater, and zone III lower, in non-breeding females compared to the PregCont group. The thickness of zone IV was significantly lower in the PregHMB group compared to the PregCont group. Other changes were not observed.

Total articular cartilage thickness was significantly greater in the PregHMB group compared to the PregCont group. Zone I and II was thinner in the non-breeding females compared to the PregCont group. Zone III was thicker in the PregHMB and NonPreg compared to the PregCont group.

### 2.5. Analysis of the Content of Thin Collagen and Proteoglycan

The content of thin collagen fibers (%) in the trabeculae, compact bone, and articular cartilage of femora from control pregnant females (PregCont), pregnant HMB-supplemented females (PregHMB), and non-pregnant females (NonPreg) is presented in Table 5. A significantly greater content of thin collagen fibers in the epiphyseal trabeculae was found in the PregHMB group compared to the PregCont group. Metaphyseal trabeculae thin collagen content was significantly lower in the group of non-breeding females and that belonging to the PregHMB group compared to the PregCont group. The content of thin collagen fibers in compact bone was significantly lower in the NonPreg group and higher in the PregHMB group compared to the PregCont group. There were no significant differences between groups in the content of thin collagen fibers in articular cartilage.

**Figure 2:**
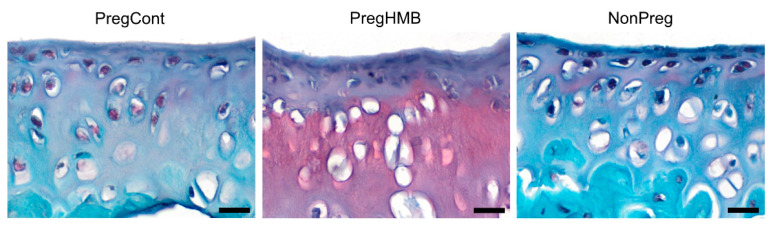
Representative images of Safranine O-stained sections of articular cartilage from femora of pregnant (control and supplemented with β-Hydroxy β-methylbutyrate (HMB)) and non-pregnant female spiny mice. PregCont—control pregnant females (not receiving HMB); PregHMB—pregnant HMB females (receiving HMB at a dose of 0.02 g/kg b.w. during the middle trimester of pregnancy); NonPreg—non-pregnant females. Scale bars represent 50 µm.

Safranin O staining showed a difference in the content of proteoglycans (based on the intensity of the pink-rose color) between the articular cartilage of pregnant females from the control and HMB-supplemented groups and non-pregnant females (Figure 2). A very low proteoglycan content (evidenced by the very low/almost absent pink color) was observed in the whole articular cartilage, but it was particularly visible in the transitional zone (zone II), with a weaker pink color observed in the deep zone (zone III) of pregnant females without HMB supplementation and in the non-breeding females.

## 3. Discussion

Human development is a process that begins at fertilization and ends when the body reaches biological maturity. The most intense period of development in humans occurs during the first two decades of life. During this time, significant changes in body structure and composition, as well as in metabolic, physiological and immune functions take place. The rate at which these processes take place slows down with age. Somatic development, including an increase in body size and weight, is very important [28]. With linear growth and weight gain, maturation plays a major role in the physical development of every human being [28]. Bone is not a metabolically static organ and undergoes a continuous process of remodeling throughout an individual’s lifetime. After reaching peak bone mass, the process of remodeling does not stop; instead, bone-forming processes slow down, and a gradual decrease in bone mineral density (BMD) begins [29,30,31,32]. The processes involved in bone tissue remodeling, in both men and women, are under strict hormonal control, with the sex hormones (estrogens in women) playing an important role. In women, estrogen levels fluctuate significantly throughout life. For this reason, women are more likely to experience significant changes in the rate of bone turnover, which affects peak bone mass and the rate of bone loss during menopause, as well as the incidence of osteopenia and osteoporosis with fractures. Osteoporosis is commonly associated with old age; however, it has been shown to occur in younger people as well, although to a lesser extent than that observed in elderly people. Pregnancy has also been shown to influence the quality of bone tissue in women and the occurrence of osteoporosis [29,30,31]. Pregnancy and lactation are very specific, although physiological, states in a woman’s life. A physiological pregnancy is defined as a single intrauterine pregnancy that is uncomplicated, in a healthy woman with an unburdened history, where delivery takes place after the 37th week of pregnancy [33]. During pregnancy, nutrient requirements increase, including that of calcium. A large supply of calcium is required, specifically during the third trimester of pregnancy. From the first trimester, a pregnant woman accumulates calcium. The main processes which ensures calcium supply to the fetus include increased intestinal calcium absorption, resorption of the maternal skeleton, and reduced urinary calcium excretion [34,35,36]. Some clinicians suggest that increased calcium absorption in the gastrointestinal tract may be not sufficient for the pregnant woman and the growing fetus, and therefore may lead to increased maternal bone resorption and bone loss. Bone turnover in non-pregnant females is different from that observed during pregnancy. Additionally, the bone turnover of a somatically and physiologically mature pregnant woman is probably different from that of a pregnant woman who is somatically immature. The duration of pregnancy also has a significant impact on bone mass. Pregnancy, due to the high metabolic demand on a woman, has a significant impact on the maternal processes of growth and development, and in the absence of full somatic maturity, it may lead to inhibition of maturation in the mother, which would be characterized by a much lower peak bone mass. Pregnancy-related osteoporosis is also generally associated with pregnancy- and lactation-associated osteoporosis (PLO). It is a rare form of osteoporosis that is severe and has an ambiguous cause. Severe back pain that interferes with basic life functions may manifest itself at the end of pregnancy (usually in the third trimester) or in the first few months after delivery, and may be accompanied by worsening thoracic kyphosis and a reduction in height. It is most common in the first pregnancy and rarely appears for the first time in subsequent pregnancies. These ailments are a consequence of low-energy vertebral fractures, which occur as a result of a decrease in the mineral mass of bone tissue and mechanical stresses during pregnancy [37]. Osteoporosis has been reported in pregnant women, and it has been suggested that this pathological condition is a transient failure that usually occurs in women with pre-existing osteopenia [38,39,40].

Despite numerous diagnostic methods (densitometry, computed tomography, evaluation of the level of bone turnover markers in serum and urine) that are used in the diagnosis of bone metabolism disorders and as markers of independent risk factors for osteopenic and osteoporotic fractures, which allow for the assessment of the rate and intensity of changes in bone tissue turnover, there are no satisfactory data on the metabolism of bone tissue and the basic histomorphometric parameters of trabecular bone during pregnancy. There is also a lack of data on the effect of HMB supplementation during pregnancy on bone metabolic activity. For this reason, animal research is so important in the process of implementing any supplementation in humans.

In the present study, no statistically significant differences in body weight were found between groups of females supplemented with HMB and those not receiving HMB supplementation, as reported previously [41], which is also in agreement with some other studies conducted on pigs [42,43,44]. Our data also showed that pregnancy did not affect the body weight of the mice immediately after delivery as compared with non-breeding females. It should be noted that our females were somatically mature at the beginning of pregnancy. Thus, further studies to confirm our preliminary results regarding lean body mass or the body weight gain of the pregnant and non-breeding females are warranted.

To our knowledge, there is no data concerning HMB supplementation in pregnant women. Available data from animal studies have demonstrated no difference in the body weight of females supplemented with HMB (at a dose of 0.02 g/kg body weight) during pregnancy and control groups [45,46]. Based on the results obtained from the current study, we can conclude that HMB supplementation did not affect the duration of pregnancy or the average number of newborns born [16,41,45,46,47]. However, previous studies with HMB supplementation in pregnant animals have shown an increase in body weight of the offspring from supplemented females [16,45]. Many previous studies on the perinatal period confirm the significant impact of HMB supplementation on body weight and composition, which is a result of its anti-catabolic effects [48,49]. However, a decrease in proteolysis, rather than an increase in protein synthesis, seems to be the underlying mechanism by which HMB increases lean body mass gain. In addition, changes in protein synthesis only seem to occur in specific tissues [50].

In the current study, the weight of femora from pregnant females was also not significantly different between the two groups, regardless of HMB supplementation. However, the group of non-breeding females had significantly lighter femora compared to the females that gave birth. Also, bone length was significantly greater in the groups of pregnant females, regardless of HMB supplementation, compared to the groups of non-breeding/non-pregnant females. The differences observed may be a consequence of the different rate and intensification of bone tissue metabolism in pregnant vs. non-pregnant females. In humans, bone turnover in physiological and uncomplicated pregnant women is more intense than that in non-pregnant women. In women with PLO, bone loss is more intense, especially in cases where bone density was lower than normal before pregnancy, and bone demineralization accelerates during the perinatal period. The risk for clinically significant fractures (low-energy fractures) is related not only to the bone structure, but also to the BMD and quality of the bones, which is the main clinical problem in PLO. A clinical case involving a 33-year-old woman with PLO showed changes in the structure of her vertebral bones, without any other significant changes in the rest of her body [37]. These changes could possibly be attributed to the patient not reaching the peak bone mass expected for her age and race, which usually occurs in the spine at around 30 years of age [32].

The study of typical changes in bone density during pregnancy is difficult, due to concerns about exposing the fetus to the radiation related to the measurement of dual energy X-ray absorptiometry (DXA). To avoid the negative effects of DXA on the fetus, only the density of peripheral skeletal sites is measured during pregnancy, or the bone density level is determined using baseline measurements taken before pregnancy and then control measurements performed shortly after delivery. Archival DXA results obtained during pregnancy and lactation show a decrease in BMD in the distal part of the forearm by about 2.0% during pregnancy, and by about 5.3% after 3 months of lactation, in the lumbar spine [51]. Another study, which was carried out in 16 pregnant women, showed increased bone turnover and a decrease in BMD of certain bone tissue during pregnancy. Total BMD measurements were performed in the women at 16, 26, and 36 weeks of pregnancy and then again immediately after delivery. Postpartum BMD was increased in the humerus (2.8%, *p* < 0.01) and limbs (1.9%, *p* < 0.01) of the women, but decreased in the pelvis (23.2%, *p* < 0.05) and spine (24.6%, *p* < 0.01) compared to values obtained during pregnancy [52].

BMD in the present study was determined for the whole bone and was not significantly different between groups, irrespective of pregnancy and HMB supplementation. This does not mean that in some regions BMD could not be significantly various. Thus, the lack of difference in BMD in particular parts of bone cannot be concluded based on this study. However, the fact that in the PregCont group, it was 3.04 g/cm^2^, whereas in the PregHMB and NonPreg BMD, it was 2.92 g/cm^2^ and 2.95 g/cm^2^, respectively, suggests clinical importance. The lack of statistical significance surely arises from one of the biggest limitations of our study: the limited number of females. However, the results of this study may seem unreliable. Despite femur (the longest bone in humans) fractures being one of the most common injuries in humans, with a head or neck fracture occurring more often than a shaft fracture, pathological bone fractures, often caused by minor trauma in PLO, tend to occur in the thoracic or sacral segments of the spine in vertebrates. This is probably due to the rapid rate of bone tissue metabolism during pregnancy and the physiological changes that occur in the skeleton during this period—that is, greater changes in the trabecular bone compared to compact bone, the imbalance between the rapid rate of bone turnover and the insufficient amount of material for mineralization, as well as the physical activity of the mother in the postpartum period (the need to carry the baby, etc.) and the associated traumas.

Our results showed changes in stiffness, which were higher in the group of pregnant females supplemented with HMB during pregnancy, and lower in the group of non-breeding/non-pregnant females. Bone strength, in terms of yield and ultimate force, both in HMB-supplemented pregnant and in non-breeding/non-pregnant females was not different from that of the control pregnant females. Thus, an even more noteworthy finding from our study was changes in the geometric values. It means that changes in stiffness are directly related to changes in bone geometry, which changed in pregnant animals and was additionally amplified by HMB supplementation. Spiny mice have a much longer gestation period compared to other laboratory animals (which is still much shorter compared to that of humans) and therefore a much longer duration of HMB supplementation; however, their rate of bone metabolism is also somewhat different compared to that of humans. Based on these facts, further research is needed.

The skeletal system is made up of numerous bones of different shapes and lengths, but all with the same internal structure. Understanding the geometry of the bones, as well as their strength, is essential to understanding the mechanisms involved in bone remodeling that change not only with age, but also during pregnancy. Bone geometry refers to the size and shape of the bone, while bone microarchitecture refers to their spatial arrangement (spatial position, thickness, and separation of the trabeculae). The macroarchitecture of the bone refers to its shape, the distribution of trabeculae, and the variable thickness of the compact bone. Thus, the mechanical and geometrical parameters are indicative of the bone’s resistance to stress during load-bearing exercises, for example when walking.

Results from the analysis of the geometrical parameters of the femora from the spiny mice in the current study showed that the lowest geometrical parameter values were observed in the non-breeding/non-pregnant females, indicating that the femora from non-breeding/non-pregnant females were not yet fully geometrically mature. These bones had lower cross-sectional area of the bone shaft, which is calculated based on the external and internal diameters of both bone dimensions. A significant increase in the bone marrow cavity and a change in the thickness of the femoral shaft were observed in femora from the pregnant females supplemented with HMB during pregnancy. Taking into account the positive changes in geometry of femora from females in this group, it can be concluded that HMB supplementation during pregnancy has a protective effect on bone metabolism. In addition, the qualitative aspect of this approach should also be taken into account. Observations were made regarding the bone morphology and geometry of the three groups of females, and further studies are warranted to investigate this aspect.

Understanding the mechanical and structural parameters of bone tissue is a necessity for the theoretical analysis of physiological bone functions. Due to its construction, the long bone is adapted to carry significant loads. The cross-section of the femoral shaft is similar to a hollow cylinder. Moreover, the mechanical strength of long bones is a result of the properties of the tissues from which the bone is made. Compact connective tissue consists of similarly oriented and strongly packed fibers, with high strength and elasticity, which results from the high content of collagen. Organic components determine the flexibility of the bone, including minerals that ensure its stiffness. The structural features of bones that affect the various mechanical parameters assessed include the multi-component structure of bone tissue with different morphology, which allows for the transfer of significant stresses and ensures significant bone ductility; and the multi-level structure of bone tissue, including the structure of the collagen fibers, which increases the tensile and compressive strength of the bone. Moreover, bone tissue is characterized by considerable adaptability to changing stress conditions. However, one should be reminded that all these components which influence the mechanical properties of bone are dependent on age (the amount of collagen decreases with age), bone geometric features, and load [53]. The mechanical parameters of bones differ depending on the area of collection of the test sample and its cross section. This differentiation disappears with age, which results from an increase in the degree of mineralization of the skeleton and adaptation to mechanics and loads. The value of Young’s modulus varies for bone tissue from long bones, depending on the location around the perimeter, as well as along the bones [53]. The degree of mineralization significantly affects the mechanical properties of the bone. High mineralization increases bone fragility, and low mineralization causes an increase in the force necessary for fracture and a consequent increase in deformation. With age, when cortical bone loss occurs (changes observed in CI and MRWT), the susceptibility of the bone shaft to fractures increases, which is also observed in humans [54,55].

In the current study, significant differences were observed in material parameters of femora between pregnant females and non-breeding/non-pregnant females. The material parameters were calculated based on equations linking the force values with the geometric parameters of the bone (linear dimensions, bone mass distribution in the cross-section). Femora from pregnant females, in both groups, were clearly heavier and longer than those of the non-pregnant females, and the cross-sectional areas of their bones were characterized by higher values for cross-sectional moment of inertia, which is one of the factors determining the stress values (elastic stress, ultimate stress) [56]. The group of pregnant females supplemented with HMB during pregnancy showed an improvement in the geometric (shaft cross-sectional area, mean relative wall thickness, cortical index), as well as mechanical and material parameters of femora, which are directly related to the susceptibility of the bone to elastic deformation (i.e., stiffness (a bone structural feature) and the Young’s modulus (a bone material property)) [53,56,57]. The results obtained following analysis of the bone geometric and mechanical properties, which showed an improvement in selected parameters in the group of pregnant females supplemented with HMB during pregnancy, are consistent with those of a previous study, which showed an improvement in the mechanical and geometric properties, as well as BMD of long bones in primiparous and multiparous minks supplemented with HMB (in a daily dose of 0.02 g/kg b.w.) during pregnancy [46]. This previous study showed for the first time that HMB supplementation in pregnant females positively affects bone density and the mechanical and geometric properties of maternal bones after delivery, and that the changes are more visible in the humerus than in the femur. Additionally, all of these bone improvements observed in the mother’s bone occur simultaneously with the same improvements observed in their offspring [46].

As previously mentioned, the overall structure and mechanical properties of bones are influenced by their hierarchical structure. Although the densitometric analysis in the current study showed no difference in BMD between groups, the histomorphometric analysis showed significant differences in the microarchitecture of trabecular bone in the distal epiphysis and metaphysis of the femora. HMB administration during pregnancy had a positive effect on relative bone volume, thickness, and number of trabeculae; however, a reduction in the trabecular space was observed. Although HMB supplementation had no effect on relative bone volume of the femoral neck, more numerous but thinner trabeculae were observed. This result is very important from the point of view of femoral neck fracture. The number of individual trabeculae at any given bone site is variable and likely determined through a relationship between trabecular size (thickness of trabeculae) and number. The trabeculae number is more important than the size of individual trabeculae when considering the overall mechanical competence of a trabecular bone, because the trabecular number has a greater effect on connectivity; therefore, the loss of trabeculae (through resorption) has a greater effect (even 2–3 times greater) on weakening the structure than does thinning of trabeculae [58]. In women, trabecular bone loss in the femoral neck region increases with age. It is likely that the increased loss of bone mass in this area in women, combined with fractures that occur in older age, are due to increased bone loss and failure to reach peak bone mass, as well as the number of pregnancies and periods of lactation the woman undergoes [59]. Our study is lacking in bone labeling, which includes dynamic parameters of bone cell function. Despite the lack of these results, the clinical importance of this finding is vast. Clinicians caring for pregnant women should pay special attention to BMD of vertebrae, as the bone loss in this part of the skeleton is key in pregnancy- and lactation-associated osteoporosis, rather than the BMD of femur, which can be complicated by trabecularization of the compact bone. This aspect should be investigated further.

Longitudinal bone growth occurs in the epiphyseal cartilage, which consists of different zones characterized by varying morphology and function. The current study showed that pregnancy and HMB supplementation had no effect on the overall thickness of the epiphyseal cartilage. Pregnancy, despite its physiological course, disrupts the rate and intensity of bone tissue synthesis. Both processes, bone synthesis and resorption, were altered during pregnancy in the mice in the current study, as indicated by the mechanical testing, QCT, and histomorphometric analyses performed. In the pregnant females that were not supplemented with HMB, bone resorption was probably more intensive, whereas the opposite result was observed in the group of females supplemented with HMB during pregnancy, where bone synthesis was improved by HMB supplementation. These observations are in agreement with a previous study in which a slight increase in the concentration and activity of bone turnover markers (osteocalcin, alkaline phosphatase, C-telopeptide of type I collagen) associated with the presence and healing of fresh bone fractures were observed in some women with PLO [60,61,62]. Moreover, bone biopsies of some PLO patients have indicated increased resorption or decreased activity of osteoblasts [40,63,64].

Analysis of the content of thin (immature) collagen fibers in the trabeculae, compact bone, and articular cartilage of femora of the spiny mice used in the current study supports the suggestion of the intensification of bone metabolic processes, especially in females supplemented with HMB during pregnancy, which is consistent with available data in the literature. However, as previously mentioned, there is currently no available literature on HMB supplementation in women, and the available information relates to experimental animal studies. The data presented by Tomaszewska et al. [13] show that maternal HMB supplementation contributes to the growth of mature collagen fibers.

In the current study, safranin O staining of articular cartilage also showed differences in proteoglycan content (shown by differences in the intensity of the red color). Weak safranin O staining of similar intensities was observed in the articular cartilage from the groups of non-breeding/non-pregnant females and in the group of control pregnant females not supplemented with HMB during pregnancy. The highest content of proteoglycans was found in the articular cartilage from the group of pregnant females supplemented with HMB during pregnancy. Proteoglycans are synthesized and released into the matrix by chondrocytes, and the concentration of proteoglycans is an indication of the metabolic capacity of the cartilage cells. Chondrocytes, in addition to proteoglycans, produce type II collagen [65]. The composition and proportions of the basic components of articular cartilage and their distribution in individual zones determine the viscoelastic properties and hydrodynamic functions of the articular cartilage [66]. Proteoglycans are the most abundant component of cartilage matrices, which are made up of keratin sulfate, chondroitin sulfate, and hyaluronic acid [67]. Proteoglycans are characterized by their specific ability to bind water, which is a primary determinant of the elastic properties of articular cartilage. In humans, there is a loss of water in the articular cartilage with age (as a result of reduced chondrocyte metabolism and a decrease in proteoglycan synthesis), which is manifested as an increased susceptibility of the articular cartilage to damage, especially within the surface zone. The surface zone has the ability to disperse and decrease the forces acting on the articular surface. Damaged cartilage has been shown to deteriorate. Progressive processes of atrophy in the articular cartilage tissue change the strength of the forces acting in the joint and lead to changes in bone geometry. The change in geometry, of course, is dependent on the intensification and rate of bone tissue metabolic processes, which depend on the activity of the basic bone cells, the osteoblasts and osteoclasts [67,68].

There is no information in the available literature on the effects of HMB supplementation on human bone tissue during pregnancy, and there is only one animal study on this topic [13]. The results of the current study are therefore unique and suggest that administering HMB to pregnant females has a very positive effect on their bone tissue. A previous study in piglets highlighted some adverse effects of HMB supplementation during pregnancy, due to the possible effects of the supplement on the newly developing organism. These previous results suggest that prenatal exposure to HMB disturbs hormonal homeostasis, which impairs early folliculogenesis in piglets [69]. Other studies have shown a negative effect of HMB on hippocampal function in male offspring [14]. Whether the adverse effects of HMB supplementation during pregnancy are due to the dose of HMB administered or are a result of the stage of pregnancy during which supplementation occurs still remains to be determined. However, the introduction of HMB as a supplement in humans during pregnancy requires, as shown by a small number of animal studies, a great deal of further experimentation for the safety of the fetus.

The present study has some limitations, such as the lack of methods allowing for the determination of osteoblast/osteoclast activity or rate in bone formation, and the lack of biochemical analysis related to bone mineralization, macroelements intestinal absorption, and transfer of macroelements or of HMB (per se or its metabolites) from mother to fetus. Although the general mechanism of HMB in metabolism is known, it must be highlighted that pregnant and fetus bone metabolisms, and their relationships in the case of HMB supplementation, are unknown. For this reason, in our opinion obtained results can be clinically helpful, especially as animal studies are irreplaceable research tools supporting medical knowledge in obstetrics, particularly skeletal pathophysiology. Further studies involving HMB supplementation in pregnancy are needed.

## 4. Materials and Methods

The experiments were performed in accordance with the Polish legal requirements, under the license of the II Local Ethical Committee in Lublin, Poland (No. 55/2013 and 8/2014) and in accordance with EU Directive 2010/63/EU.

### 4.1. Animals and Experimental Design

The study was conducted according to the guidelines of the Declaration of Helsinki, and approved by the II Local Ethics Committee in Lublin, Poland (protocol 55/2013 and 8/2014).

The spiny mice were bred under laboratory conditions at the Maria Curie-Skłodowska University in Lublin, Poland. Clinically healthy, mature, 6-month-old (*n* = 30) female spiny mice (*Acomys cahirinus*) were used in this study. The study included both non-pregnant (*n* = 10) and pregnant (*n* = 20) females, divided into three groups (*n* = 10 each), as detailed below. The females were mated naturally with healthy fertile males (at a ratio of 1 male to 1 female). The precise mating time cannot be determined in this species due to the lack of visible vaginal plugs; therefore, a postpartum estrus exhibited by the spiny mouse within 24 h of giving birth was used to determine gestational ages. Only females in their second pregnancy, approximately 6 months of age at the time of delivery, were used in the current study.

Female spiny mice were randomly divided into the following groups: pregnant control females (group PregCont; *n* = 10, not receiving HMB); pregnant HMB females (group PregHMB; *n* = 10) receiving HMB at a dose of 0.02 g/kg b.w., between the 13th and 26th days of pregnancy (middle trimester); and non-pregnant females, who had never given birth and were not pregnant at the time (group NonPreg; *n* = 10).

All spiny mice were individually housed in standardized cages (Velaz, Praha, Czech Republic) and were kept under identical conditions, at a constant temperature of 22 ± 2 °C, humidity of 55–60%, and a controlled 12 h/12 h light/dark cycle, with an airflow speed not exceeding 0.3 m/s. The mice had free access to water and were fed a standard laboratory rodent’s diet, formulated to meet nutritional requirements specified in the AIN-93M directive [70], in the pellet form. Animals were fed twice a day (at 08:00 and 12:00 a.m.). The total amount of food consumed by the females was estimated before the start of the experiment and was calculated as 15 g of feed per day (three pellets). In the morning, between the 13th and 26th day of pregnancy, the pregnant females from the PregHMB group received one pellet with HMB, while the pregnant control females received feed alone. Pregnant females from group PregHMB were weighed daily to determine HMB dose. At 12:00, the remaining part of the feed (two pellets) was given to all females.

Commercially available beta-hydroxy beta-methylbutyrate (HMB), in the form of powder (purity ≥ 99%) (Lonza, Basel, Switzerland) was used in the study. The dose of HMB used (0.02 g/kg b.w.) was based on the literature and previous studies [17,42,45,48,71,72,73,74,75].

Females from all experimental groups, as well as the newborn pups from females in groups PregCont and PregHMB were euthanized by CO_2_ inhalation. To accomplish the 3R’s rule, tissue samples received from pups were used in another study [14]. Immediately after euthanasia, femora were carefully dissected out of the mice, cleaned of adhering tissues, individually packed, and stored frozen at −20 °C until they were examined.

### 4.2. Densitometry and Micro Computed Tomography Analysis

After thawing overnight at 7 °C, right femora were weighed and measured for length, and the whole-bone mineral density (BMD) was measured using a Lunar iDXA densitometer (GE, Madison, WI, USA). A scan of the whole bone was performed using the “small animal” mode of the enCORE^®^ software (ver.17.0, GE, Madison, WI, USA) and a special pad for “small animal” scans, provided by the producer of the densitometer, which was used to eliminate measurement artifacts and to immobilize the bones so that all the bones were scanned in the same plane.

Next, X-ray computed tomography analysis was performed using a Nanotom 180S device (GE Sensing & Inspection Technologies GmbH, Wunstorf, Germany; rotation step: 0.3 deg, scan resolution: 11 µm; X-ray source voltage: 140 kV; X-ray source current: 250 µA; Cu filter: 0.3 mm; rotation: 360°; rotation step: 0.24°). Reconstruction was performed using DatosX 2.0 software (GE Sensing & Inspection Technologies GmbH, Wunstorf, Germany) and 16-bit, grey-level 3D images were generated. Image analysis was performed using VG Studio Max 2.0 (Volume Graphics GmbH, Heidelberg, Germany) and ImageJ (U.S. National Institutes of Health, Bethesda, MA, USA) and Avizo 9 (FEI, Hillsboro, OR, USA) software [76].

Regions of interest (ROI) were placed at (i) the middle part of the lateral condyle (sagittal section located perpendicularly to the articular surface), (ii) the femoral mid-shaft (transverse section), and (iii) the trabecular bone of the femoral neck (transverse section below femoral head). Median filtering with kernel size 2 px was applied to minimize the noise before thresholding the images with the IsoData algorithm. Images were analyzed for geometry (mid-shaft scan) and trabecular bone measurements using BoneJ plugin for ImageJ software [77]. Bone mid-shaft geometric traits (cross-sectional area, cortical index, mean relative wall thickness, cross-sectional moment of inertia, and radius of gyration) were determined from X-ray computed tomography scans, on the basis of measurements of cortical bone cross-sectional diameters [56,78]. The measurements included trabecular bone volume fraction (BV/TV, %), trabecular thickness (Tb.Th, µm), trabecular space (Tb.Sp, µm), trabecular number (Tb.N, 1/mm), and specific bone surface (BS/BV, %) [79].

### 4.3. Mechanical Testing

Left femora were subjected to the three-point bending test on a universal testing machine (Zwick-Roell 005, Zwick-Roell GmbH & Co., Ulm, Germany). Bones, placed on two support points, positioned apart at 40% of total bone length, were loaded in bending at the midpoint of the bone mid-shaft with a constant load rate of 2 mm/min until fracture. On the basis of recorded load-deformation curves, bone structural properties were determined: the yield force was determined as maximal force under elastic (reversible) deformation, and the ultimate force as the force causing bone fracture; stiffness was measured as the slope of the elastic part of the load-displacement curve; and bending moment was calculated using an appropriate equation [56].

Bone material properties (intrinsic properties of the bone tissue) were calculated using data obtained in the three-point bending test and determined bone mid-shaft geometric traits. They included Young’s modulus of elasticity (describing bone bending resistance), fracture strain (describing the relative deformation under fracture load), elastic stress (describing the elastic strength) and the ultimate stress (equal to the maximum stress bone can withstand in bending before fracture) [57,78].

### 4.4. Histomorphometry

After the bending test, the distal epiphysis was cut and fixed in phosphate-buffered 4% (*v*/*v*) paraformaldehyde (pH 7.0) for 48 h, decalcified in a 10% (*w*/*v*) ethylene diamine tetra acetic acid (EDTA) solution (pH 7.4) for 3–4 weeks on a shaker at 4 °C, dehydrated through in an ascending ethanol series (30–70%, *v*/*v*), fixed with nonpolar Ottix Plus and Ottix Sharper solvents (DiaPath, Martinengo, Italy), and embedded in paraffin blocks. Sagittal sections (5 μm thickness) of the middle part of the lateral condyle were obtained using a microtome (HM360, Microm, Walldorf, Germany) [80].

The sections were stained with Safranin O to evaluate proteoglycan distribution in articular cartilage, with Goldner’s trichrome to evaluate basal morphology of the growth plate and articular cartilage, and with Picrosirus red (PSR) to evaluate the distribution of immature (thin) and mature (thick) collagen fibers [81]. The sections were photographed in brightfield (Safranin O, Goldner’s) and polarized (PSR) light, using a CX43 microscope (Olympus, Tokyo, Japan) equipped with a UC50 digital camera (Olympus, Tokyo, Japan). Images of the Goldner’s stained sections were examined for determination of total thickness of the growth plate cartilage, as well as the thickness of the main zones: the resting zone (I), the proliferative zone (II), the hypertrophic zone (III), and the calcified zone (IV) [82]. For the articular cartilage, total thickness and the thicknesses of the superficial (I), middle (II), and deep (III) zones were measured [78]. Immature collagen content was quantified as the percentage of total collagen observed in PSR-stained sections, using the pixel counting method. For all analyses, three slides/bone with 20 μm separation were analyzed. The images were analyzed using ImageJ [83] and CellSens (Olympus, Tokyo, Japan) image analysis software.

### 4.5. Statistical Analysis

Data are reported as mean ± standard deviation (SD). All statistical procedures were performed using Statistica software (v. 13.0, TIBCO Software Inc., Palo Alto, CA, USA). Statistical analysis was done to examine the effect of pregnancy (PregCont vs. NonPreg groups) and HMB supplementation (PregCont vs. PregHMB groups) for measured traits. All variables were examined for normality (Shapiro–Wilk test) and homogeneity of variance (Levene test) and were analyzed using a two-tailed Student’s *t*-test, *t*-test with Welch’s correction when normally distributed data lacked equal variances, or a non-parametric Mann–Whitney U test when there was a lack of normal distribution. In all analyses, a *p* ≤ 0.05 was considered statistically significant.

## 5. Conclusions

In summary, the current study based on a spiny mice model has shown that dietary HMB supplemented in the second trimester of pregnancy modulates mothers’ bone metabolism.

## Figures and Tables

**Table 1:** Osteometric, mechanical, and densitometric parameters of femora from pregnant (control and supplemented with β-Hydroxy β-methylbutyrate (HMB)) and non-pregnant female spiny mice.

Item	PregCont	PregHMB	NonPreg
Weight, g	0.16 ± 0.02	0.15 ± 0.01	0.13 ± 0.02 ^#^
Length, mm	20.52 ± 0.24	20.31 ± 0.26	18.88±0.34 ^#^
BMD, g/cm^2^	3.04 ± 0.18	2.92 ± 0.30	2.95 ± 0.22
F_max_, N	23.63 ± 5.58	24.17 ± 3.25	21.48 ± 2.84
F_yield_, N	19.25 ± 1.36	18.58 ± 2.77	17.51 ± 2.36
S, N/mm	64.51 ± 5.34	81.70 ± 2.45 *	41.1 5± 5.45 ^#^
M, N⋅m	48.37 ± 1.94	48.95 ± 2.29	40.56 ± 6.75 ^#^
CSMI, mm^4^	0.34 ± 0.06	0.32 ± 0.07	0.20 ± 0.01 ^#^
CSA, mm^2^	1.45 ± 0.09	1.56 ± 0.04 *	1.17 ± 0.05 ^#^
MRWT	0.44 ± 0.05	0.54 ± 0.03 *	0.45 ± 0.05
CI, %	30.30 ± 0.11	34.61 ± 0.41 *	31.07 ± 2.44

Data are mean values ± SD (*n* = 10 in each group). PregCont—control pregnant females (not receiving HMB); PregHMB—pregnant HMB females (receiving HMB at a dose of 0.02 g/kg b.w. during the middle trimester of pregnancy); NonPreg—non-pregnant females. BMD—bone mineral density; F_max_—ultimate force; F_yield_—yield force; S—stiffness, M—bending moment; CSMI—cross-sectional moment of inertia; CSA—cross-sectional area; MRWT—mean relative wall thickness; CI—cortical index. Statistical significance: * *p* ≤ 0.05 (vs. control group of PregCont), ^#^ *p* ≤ 0.05 (vs. control group of PregCont).

**Table 2:** Material parameters of femora from pregnant (control and supplemented with β-Hydroxy β-methylbutyrate (HMB)) and non-pregnant female spiny mice (*Acomys cahirinus*).

Item	PregCont	PregHMB	NonPreg
Young’s modulus, MPa	1862 ± 204	263 9± 112 *	1937 ± 74
Ultimate strain, %	5.35 ± 1.25	3.54 ± 0.82 *	6.19 ± 2.15
Elastic stress, MPa	92.67 ± 4.8	89.56 ± 6.89	120.12 ± 4.80 ^#^
Ultimate stress, MPa	113 ± 4	115 ± 3	148 ± 3 ^#^

Data are mean values ± SD (*n* = 10 in each group). PregCont—control pregnant females (not receiving HMB); PregHMB—pregnant HMB females (receiving HMB at a dose of 0.02 g/kg b.w. during the middle trimester of pregnancy); NonPreg—non-pregnant females. Statistical significance: * *p* ≤ 0.05 (vs. control group of PregCont), ^#^ *p* ≤ 0.05 (vs. control group of PregCont).

**Table 3:** Osteometric, mechanical, and densitometric parameters of femora from pregnant (control and supplemented with β-Hydroxy β-methylbutyrate (HMB)) and non-pregnant female spiny mice.

Item	PregCont	PregHMB	NonPreg
**Distal epiphysis**			
BV/TV, %	30.03 ± 2.74	35.28 ± 3.84 *	27.14 ± 0.81 ^#^
Tb.Th, µm	125.16 ± 17.71	56.15 ± 9.01 *	74.87 ± 9.61 ^#^
Tb.Sp, µm	220 ± 23	162 ± 30 *	286 ± 34 ^#^
Tb.N, 1/mm	4.03 ± 0.27	5.59 ± 0.48 *	3.75 ± 0.39
BS/BV, %	15.97 ± 3.89	11.23 ± 2.40 *	14.97 ± 1.92
**Distal metaphysis**			
BV/TV, %	31.41 ± 2.86	35.96 ± 2.16 *	29.41 ± 2.72
Tb.Th, µm	47.20 ± 8.73	45.27 ± 6.66	68.03 ± 16.23 ^#^
Tb.Sp, µm	125 ± 19	101 ± 13 *	168 ± 27 ^#^
Tb.N, 1/mm	6.81 ± 1.31	8.09 ± 0.91 *	4.36 ± 0.31 ^#^
BS/BV, %	9.44 ± 2.00	7.33 ± 1.09 *	13.61 ± 3.24 ^#^
**Femoral neck**			
BV/TV, %	36.76 ± 2.26	36.50 ± 4.45	42.13 ± 3.25 ^#^
Tb.Th, µm	104.9 ± 11.5	90.5 ± 12.9 *	112.7 ± 8.93
Tb.Sp, µm	228 ± 35	265 ± 55	247 ± 26
Tb.N, 1/mm	3.47 ± 0.62	4.03 ± 0.55 *	3.82 ± 0.43 ^#^
BS/BV, %	21.34 ± 1.38	18.10 ± 2.58 *	22.17 ± 2.08

Data are mean values ± SD (*n* = 10 in each group). PregCont—control pregnant females (not receiving HMB); PregHMB—pregnant HMB females (receiving HMB at a dose of 0.02 g/kg b.w. during the middle trimester of pregnancy); NonPreg—non-pregnant females. BV/TV—bone volume density; Tb.Th—trabecular thickness; Tb.Sp—trabecular space; Tb.N—trabecular number; BS/BV—specific bone surface. Statistical significance: * *p* ≤ 0.05 (vs. control group of PregCont), ^#^ *p* ≤ 0.05 (vs. control group of PregCont).

**Table 4:** The thickness of the epiphyseal and articular cartilage zones of femora from pregnant (control and supplemented with β-Hydroxy β-methylbutyrate (HMB)) and non-pregnant female spiny mice.

Item	PregCont	PregHMB	NonPreg
**Growth plate cartilage**			
total, µm	203 ± 31	172 ± 13 *	205 ± 15
I, µm	24.45 ± 10.92	26.33 ± 8.85	27.14 ± 8.03
II, µm	52.08 ± 10.13	51.69 ± 11.36	68.60 ± 8.96 ^#^
III, µm	46.39 ± 5.37	42.46 ± 9.66	37.27 ± 8.53 ^#^
IV, µm	48.79 ± 11.21	29.72 ± 6.91 *	58.56 ± 14.59
**Articular cartilage**			
total, µm	83.87 ± 5.69	92.13 ± 4.92 *	83.41 ± 5.41
I, µm	12.07 ± 2.55	10.57 ± 3.27	6.59 ± 1.31 ^#^
II, µm	28.94 ± 7.59	35.18 ± 10.9	21.76 ± 7.3 ^#^
III, µm	41.00 ± 2.68	48.26 ± 2.88 *	54.63 ± 2.96 ^#^

Data are mean values ± SD (*n* = 10 in each group). PregCont—control pregnant females (not receiving HMB); PregHMB—pregnant HMB females (receiving HMB at a dose of 0.02 g/kg b.w. during the middle trimester of pregnancy); NonPreg—non-pregnant females. Zones of growth plate cartilage: I—resting zone; II—proliferative zone; III—hypertrophic zone; IV—calcified zone. Zones of articular cartilage: I—superficial zone; II—middle (transitional) zone; III—deep zone. Statistical significance: * *p* ≤ 0.05 (vs. control group of PregCont), # *p* ≤ 0.05 (vs. control group of PregCont).

**Table 5:** Thin collagen content (%) in trabeculae, compact bone and articular cartilage of femora from pregnant (control and supplemented with β-Hydroxy β-methylbutyrate (HMB)) and non-pregnant female spiny mice.

Item	PregCont	PregHMB	NonPreg
Epiphyseal trabeculae	0.39 ± 0.13	1.08 ± 0.13 *	0.36 ± 0.14
Metaphyseal trabeculae	0.87 ± 0.15	0.67 ± 0.15 *	0.27 ± 0.11 ^#^
Compact bone	1.92 ± 0.67	2.82 ± 0.09 *	0.93 ± 0.06 ^#^
Articular cartilage	1.69 ± 1.02	1.57 ± 1.05	1.63 ± 0.21

Data are mean values ± SD (*n* = 10 in each group). PregCont—control pregnant females (not receiving HMB); PregHMB—pregnant HMB females (receiving HMB at a dose of 0.02 g/kg b.w. during the middle trimester of pregnancy); NonPreg—non-pregnant females. Statistical significance: * *p* ≤ 0.05 (vs. control group of PregCont), ^#^ *p* ≤ 0.05 (vs. control group of PregCont).

## Data Availability

The data presented in this study are available on request from the corresponding author.
